# Lower Dose of 5 mL of 1% Lidocaine is More Suitable than the Conventional 10 mL for Caudal Block in Transrectal Prostate Biopsy: A Retrospective Cohort Study

**DOI:** 10.1155/2024/9331738

**Published:** 2024-02-14

**Authors:** Norichika Ueda, Mototaka Sato, Shunsuke Mori, Atsuki Matsukawa, Yuta Oki, Yuma Kujime, Ryoya Mizuno, Hiromu Horitani, Tetsuya Yamamoto, Shota Fukae, Mitsuhiro Yoshinaga, Makoto Matsushita, Mai Akiyama, Satoshi Kamido, Ayako Honda, Jiro Nakayama, Norihide Tei, Osamu Miyake

**Affiliations:** ^1^Department of Urology, Toyonaka Municipal Hospital, 4-14-1 Shibahara-cho, Toyonaka, Osaka 560-8565, Japan; ^2^Department of Urology, Osaka Rosai Hospital, 1179-3 Nagasanecho, Kita-ku, Sakai, Osaka 591-8025, Japan; ^3^Department of Urology, Higashiosaka City Medical Center, 3-4-5 Nishiiwata, Higashiosaka, Osaka 587-8588, Japan; ^4^Department of Urology, Ikeda City Hospital, 3-1-18 Jonan, Ikeda, Osaka 563-8510, Japan; ^5^Department of Urology, Hyogo Prefectural Nishinomiya Hospital, 13-9 Rokutanjicho, Nishinomiya, Hyogo 662-0918, Japan; ^6^Department of Urology, Minoh City Hospital, 5-7-1 Kayano, Minoh, Osaka 562-0014, Japan; ^7^Pharmaceuticals and Medical Devices Agency, 3-3-2 Kasumigaseki, Chiyoda-ku, Tokyo 100-0013, Japan; ^8^Department of Anesthesiology, Toyonaka Municipal Hospital, 4-14-1 Shibahara-cho, Toyonaka, Osaka 560-8565, Japan; ^9^Department of Urology, Suita Tokushukai Hospital, 21-1 Senriokanishi, Suita, Osaka 565-0814, Japan

## Abstract

**Objectives:**

In Japan, caudal block with 1% lidocaine is commonly used for transrectal prostate biopsy. Although 10 mL of 1% lidocaine is commonly used, the appropriate dosage of 1% lidocaine has not been studied. Our hospital routinely uses two different doses (5 or 10 mL) of 1% lidocaine for caudal block for transrectal prostate biopsy. Herein, we retrospectively evaluated the efficacy and safety of both doses of 1% lidocaine.

**Methods:**

This retrospective study included 869 patients who underwent transrectal prostate biopsy with caudal block at our hospital. The amount of 1% lidocaine was determined by the day of the week on which the biopsy was performed, and the patient voluntarily chose the day of the biopsy, unaware of the dose of 1% lidocaine used on that day. Pain, anal sphincter tonus, cancer diagnosis rate, and early complications were compared.

**Results:**

In total, 466 and 403 patients received 5 and 10 mL of 1% lidocaine for a caudal block, respectively. After propensity-score matching for patient characteristics, each group contained 395 patients. The pain score, anal sphincter tonus score, or prostate cancer diagnosis rate were not significantly different between the two groups. However, rectal bleeding was significantly more frequent and severe in the 10-mL than the 5-mL group (*p*=0.018 and *p*=0.0036, respectively). The incidence of other complications was not significantly different between the groups.

**Conclusions:**

Our results suggest that 5 mL of 1% lidocaine may be more suitable than 10 mL for caudal block during transrectal prostate biopsy.

## 1. Introduction

Prostate cancer (PCa) is the second most common malignant disease among men worldwide [1]. Since PCa is definitively diagnosed via histopathology, prostate biopsy is essential, and approximately 2.5 million prostate biopsies are performed worldwide annually [2]. More punctures in a single biopsy are associated with an improved PCa diagnostic rate, but also with more pain and complications [3]. Severe pain or complications may reduce the amount of tissue biopsied and ultimately decrease the PCa diagnostic rate [4]. Therefore, prostate biopsy should be performed with appropriate anesthesia providing good pain control and lowering complication rates.

Transrectal prostate biopsy reportedly causes pain in two instances: first, when the transrectal ultrasound probe is manipulated in the rectum and second, when the biopsy needle penetrates the prostate [5]. A variety of anesthetic methods have been reported for transrectal prostate biopsy, including intrarectal local anesthesia, periprostatic nerve block (PNB), intraprostatic local anesthesia, spinal anesthesia, caudal block, intravenous sedation, and pelvic plexus block [2]. There are many randomized control trials (RCTs) that compare the effects of these anesthetic methods, alone or in combination. However, the question of which method is the most effective way to control pain during transrectal prostate biopsy remains controversial [2]. Among the various anesthetic methods for transrectal prostate biopsy, a caudal block can theoretically reduce pain in both instances by blocking the sacral nerve, which innervates the perineum, rectum, and prostate [6]. Hence, a caudal block is commonly used for transrectal prostate biopsy, especially in Japan [7–9]. Lidocaine is commonly used for caudal block of transrectal prostate biopsy because it is cheap, safe, and easily available [10]. Lidocaine has been reported to exert its analgesic effect by blocking sodium channels [11]. The higher the concentration and volume of lidocaine, the stronger the analgesic effect. However, since lidocaine has a vasodilatory effect, the vasodilatory effect may be enhanced depending on the volume and concentration, which can cause various adverse effects [12]. In Japan, the 1% lidocaine dose customarily used in many hospitals is 10 mL. However, the appropriate dosage of 1% lidocaine for transrectal prostate biopsy has not been studied in basic or clinical experiments. In our hospital, transrectal prostate biopsy has customarily been performed with caudal block using 5 or 10 mL of 1% lidocaine. Hence, it is possible to retrospectively compare the efficacy and safety of two different doses (10 mL and 5 mL) of 1% lidocaine. Herein, we report the results of a retrospective comparison of the efficacy and safety of two doses of 1% lidocaine for caudal block for transrectal prostate biopsy.

## 2. Methods

This retrospective study included 886 patients with suspected PCa who underwent transrectal prostate biopsy with caudal block at the Department of Urology, Toyonaka Municipal Hospital, Osaka, Japan, from July 2016 to August 2022. We performed transrectal prostate biopsy with caudal block on Mondays and Fridays, with 10 mL of 1% lidocaine used on Mondays and 5 mL on Fridays. The day of prostate biopsy is determined by the patient's preference. The patients did not know the dose of 1% lidocaine they would receive. Our hospital has traditionally used two different doses of 1% lidocaine for caudal block in prostate biopsy: 5 mL or 10 mL. This was chosen by patient scheduling preference, not for the purpose of clinical research. At our hospital, the doctor in charge of prostate biopsy was determined by the day of the week. When the doctor in charge of the prostate biopsy changed, the doctor in charge informed the next doctor the amount of 1% lidocaine to be used in the prostate biopsy. As a result, these two different 1% lidocaine doses were used in caudal block in prostate biopsy for a long time, and a large number of cases were accumulated. In this study, we retrospectively extracted and discussed past clinical data. As a result, the patients were divided into two groups retrospectively based upon the dosage received, not by random assignment. This study was conducted in accordance with the principles of the Declaration of Helsinki. The research protocol was approved by the Ethics Committee of the Toyonaka Municipal Hospital (no: 20210705). Because we extracted and reviewed the historical data, we were unable to obtain patient consent in real time. The ethics committee approved the use of an opt-out consent method due of the retrospective nature of this study. Information was posted on the hospital website exclusion request for this study. The indications for prostate biopsy were a prostate-specific antigen (PSA) level >4 ng/mL or findings suggestive of PCa on digital rectal examination (DRE) or pelvic magnetic resonance imaging. Patients who could not discontinue anticoagulants and patients in whom the anesthetic needle for caudal block could not be inserted into the sacral fissure because of closure, deformity, or stenosis of the sacral hiatus were excluded.

A caudal block was performed in the treatment room with the patient in the prone position. We identified the sacral hiatus and sterilized the skin around the sacral hiatus with alcohol. A 22-G, 38-mm needle was inserted in the sacral hiatus. After confirming the absence of backflow of spinal fluid or blood, 1% lidocaine was injected. Ten minutes after caudal block, the patient was placed in the lithotripsy position and prepared for transrectal prostate biopsy.

When performing prostate biopsy, we performed DRE to assess the size, hardness, and lesion location of the prostate before puncturing the prostate. After evaluating the prostate with DRE, the patient was asked to tighten the anus while keeping the doctor's finger inside the anus. The doctor evaluated the tone of the anal sphincter muscles to confirm whether the caudal block was working. Anal sphincter tonus was graded as 0 (complete relaxation), 1 (mild relaxation), 2 (very mild relaxation), or 3 (no relaxation). The anus was then disinfected with povidone iodine, and a rectal probe (HITACHI Aloka Medical, Hitachi, Tokyo, Japan) was inserted. Each patient underwent transrectal prostate biopsy with 12 punctures using an 18-G, 20-cm biopsy needle (BD, Franklin Lakes, NJ). Immediately after prostate biopsy, we manually inserted gauze into the needle puncture site in the rectum, and we evaluated rectal bleeding by the amount of blood that adhered to the gauze. We then determined whether manual compression was necessary to stop the rectal bleeding or whether the prostate biopsy could be completed without manual compression. Rectal bleeding was evaluated using the following rectal bleeding scores: (0) there was no or very little bleeding, and no manual compression was required; (1) manual compression was required but rectal bleeding could be stopped immediately within 2 min; (2) >2 min of manual compression was required but rectal bleeding stopped within 5 min; or (3) bleeding did not stop even after 5 min of manual pressure. At score 3, gauze was placed in the anus in all the patients. A rectal bleeding score of 1 or more was defined as having rectal bleeding, and a score of 0 was defined as no rectal bleeding. Rectal bleeding score and anal sphincter tone score were completed by the attending physician immediately after prostate biopsy. The patient self-administered the numerical rating scale (NRS) pain score of the caudal block and the prostate biopsy from 0 to 10 within 30 min after the prostate biopsy was completed. The completed scores were collected that day. The timing of the pain evaluation by patients, or anal sphincter tonus and rectal bleeding by attending physicians, did not differ significantly between individual cases.

The cancer diagnosis rate was calculated as the ratio of the number of cases in which adenocarcinoma was detected among the number of cases in which prostate biopsy was performed. Urinary retention was defined as the development of urinary retention after prostate biopsy, requiring urinary catheterization or urinary catheter placement. A urinary tract infection was defined as a fever of 38°C or higher after prostate biopsy. A vagal reflex was defined as a decrease in blood pressure accompanied with cold sweating, dizziness, or turning pale after caudal block, which spontaneously recovered with leg elevation. Hematuria as a complication was defined as persistent hematuria after prostate biopsy that did not resolve spontaneously without the use of hemostatic agents. Cases in which a small amount of gross hematuria occurred after the biopsy, but the hematuria stopped spontaneously, were defined as no hematuria as complication. Complications of the prostate biopsy were noted in the patient's medical record on the same day by the attending physician. The data on complications were collected by retrospectively reviewing medical records. At our hospital, transrectal prostate biopsy was performed on the day of admission, and patients were discharged the next day to prioritize safety. The evaluation period for complications was 14 days from the time of transrectal prostate biopsy to the date of explanation of pathology results. Regarding pain, the patient was not informed of their lidocaine dose, so the assessment of pain was blind. However, anal sphincter tonus and the presence or absence of these complications was determined by the attending physician, so the assessments of complications were not blind.

Twelve urologists skilled in caudal block and transrectal prostate biopsy performed all anesthetic and biopsy procedures. Patient characteristics were evaluated, including age, height, weight, body mass index (BMI), PSA level, and prostate volume. The PCa diagnosis rate was assessed for all patients, and clinical T stage was assessed for patients diagnosed with PCa. Complications, including rectal bleeding, urinary retention, urinary tract infection, vagal reflex, and hematuria were evaluated. In this study, no patients underwent fasting or laxative bowel preparation. Regarding antibiotic prophylaxis, all patients took 500 mg levofloxacin orally immediately before transrectal prostate biopsy.

### 2.1. Statistical Analysis

The data were statistically analyzed using JMP (SAS Institute, Cary, NC). Propensity-score matching was performed to match patient characteristics. Patient characteristics, pain score, rectal bleeding score, and anal sphincter tonus score were assessed using the Mann–Whitney *U* test. The chi-squared test was used to compare the PCa diagnosis rate and presence of complications. Univariate and multivariate logistic analyses were performed to identify factors associated with rectal bleeding. The Cochran–Armitage trend test was performed to analyze the relationship between rectal bleeding and the dosage of 1% lidocaine. Statistical significance was established at *p* < 0.05.

## 3. Results

**Figure 1:**
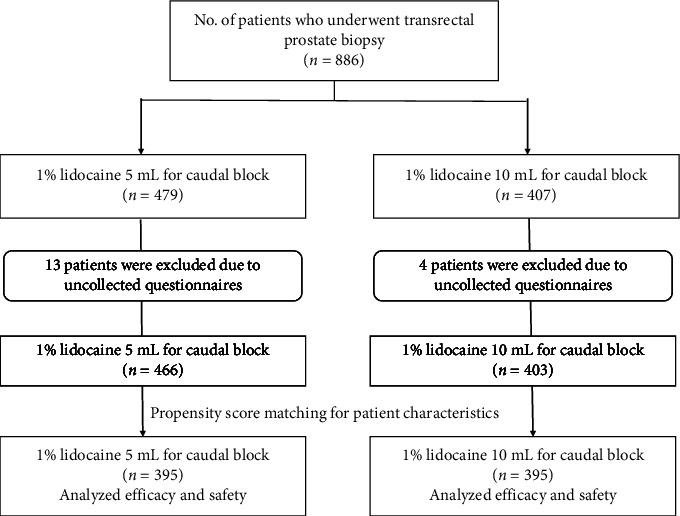
Patient flowchart.

**Figure 2:**
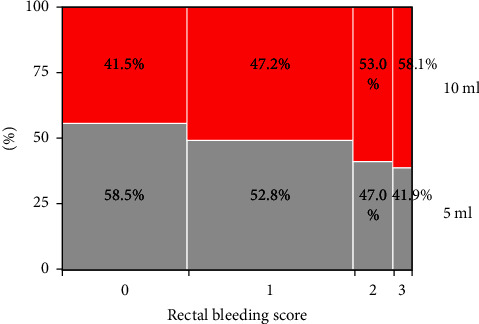
Mosaic diagram showing the relationship between 1% lidocaine dose and rectal bleeding scale. The Cochran–Armitage trend test revealed that the frequency of rectal bleeding was significantly higher with a higher dose of 1% lidocaine in the caudal block (*p*=0.0036).

Of 886 total patients who underwent transrectal prostate biopsy with caudal block during the study period, 869 were included and 17 patients were excluded as they did not complete the questionnaire (collection rate: 98.1%). In total, 466 and 403 patients received 5 mL and 10 mL of 1% lidocaine, respectively (Table 1). To adequately address potential confounding factors of patient characteristics that could influence the results, we performed analyses using propensity-matched scores. As a result of propensity-score matching for patient characteristics, the number of patients in each group was 395 (Figure 1). There were no significant between-group differences in age, height, weight, BMI, PSA level, or prostate volume (Table 2). Although there were no significant differences in PCa diagnosis rate, cT4 was significantly more common in the 10 mL group than that in the 5 mL group (*p*=0.006) (Table 3). The NRS pain scores of caudal block and prostate biopsy and anal sphincter tonus score did not significantly differ between the groups (Table 4).  Table 5 shows the early complications of transrectal prostate biopsy. The incidence of the rectal bleeding and rectal bleeding score was significantly higher in the 10 mL group than that in the 5 mL group (*p*=0.018 and *p*=0.0036, respectively). The incidence of other complications such as urinary retention, urinary tract infection, vagal reflex, and hematuria was not significantly different between the two groups. To identify factors associated with rectal bleeding, we performed univariate and multivariate logistic analysis and found that only the 1% lidocaine dose was a significant factor (*p*=0.019) (Table 6). To investigate the relationship between the amount of 1% lidocaine and the severity of rectal bleeding, we examined each group of rectal bleeding. As a result of Cochran–Armitage analysis, it was found that the higher the dose of 1% lidocaine, the more severe rectal bleeding tended to be (*p*=0.0036) (Figure 2 and Table 7). No patient had postbiopsy motor paralysis. All prostate biopsies were completed within 30 min of caudal block.

## 4. Discussion

We compared the efficacy and safety of transrectal prostate biopsy with caudal block between conventional-dose (10 mL) and low-dose (5 mL) of 1% lidocaine. There were no significant between-group differences in the pain scores of caudal block and prostate biopsy. However, rectal bleeding was significantly less frequent and less severe in the 5 mL group compared with the 10 mL group. The incidence of other complications and the PCa diagnosis rate were not significantly different between the groups. Therefore, these results suggest that caudal block with a low dose (5 mL) of 1% lidocaine is more appropriate for transrectal prostate biopsy than the conventional 10 mL dose. We believe that this is the first report to compare the dose of 1% lidocaine for caudal block in transrectal prostate biopsy and to report the usefulness of a lower dose.

Caudal block is a type of epidural anesthesia, where the anesthetic concentration and dose significantly affect the intensity and extent of anesthesia, respectively [13, 14]. We did not change the lidocaine concentration; therefore, our results reflected the difference in anesthetic range between the conventional 10 mL dose and the lower 5 mL dose. Since local anesthetics are administered into the epidural space of the sacral region in caudal block, a higher dose of anesthetic would enable nerve blockage to a higher level. The prostate is innervated by the S2–S4 and Th10–L2 nerves [15]. Therefore, theoretically, higher anesthetic doses may allow higher-level nerve blockages, resulting in better pain control. However, we found no significant between-group differences in pain during prostate biopsy. Thus, 5 and 10 mL of 1% lidocaine were suggested to be equally effective in blocking the higher-level thoracic-level nerves involved in prostate pain.

In order to evaluate the pain control level of caudal block for transrectal prostate biopsy of this study, we compared it with past reports of transrectal prostate biopsy using caudal block. To date, only six prospective studies on caudal block during transrectal prostate biopsy have been reported [6–9, 16, 17]. In these studies, the types, concentrations, and doses of anesthetics for caudal block differed, as did the patient characteristics and puncture number of the biopsy. Therefore, it was difficult to directly compare their results with ours. However, the study by Urabe et al. [17] was similar to ours as they performed caudal blocks with 10 mL of 1% lidocaine in Japanese patients, and the visual analog scale (VAS) pain score of the biopsy was also similar (3.0 ± 2.3) to our results. They reported that caudal block was equally effective for reducing pain with PNB and intrarectal local anesthesia [17]. Therefore, caudal block with 5 or 10 mL of 1% lidocaine in our study was considered to be as effective as in the previous reports. On the other hand, Horinaga et al. also performed caudal block with 10 mL of 1% lidocaine in Japanese patients [7]. Although they reported a VAS pain score of 2.1 ± 1.9, they concluded that 10 mL of 1% lidocaine was insufficient for caudal block during transrectal prostate biopsy due to the anatomical capacity of the sacral canal [7]. However, as there are no clinical studies comparing the amount of 1% lidocaine in caudal block for transrectal prostate biopsy, it is unclear to what extent pain control can be achieved with caudal block. In other words, the standards for pain control that should be achieved with caudal block are unclear. Currently, whether pain control by caudal block for transrectal prostate biopsy is sufficient or insufficient is determined by the attending physician. It is necessary to accumulate further research results and establish objective standards for pain control of transrectal prostate biopsy.

Anesthesia should aim for pain control while considering complications. Notably, in this study, complications, especially rectal bleeding, were significantly less frequent and milder in the low-dose group than in the conventional group. It is likely that the conventional 10-mL dose resulted in a wider range of anesthesia and blocked the sympathetic nervous system at a higher level than the lower 5-mL dose [13]. Consequently, it was suggested that in the 10-mL group, the range of peripheral vascular dilation due to vascular smooth muscle relaxation caused by the sympathetic nerve blockade became wider and blood flow into the rectum increased, thereby increasing rectal bleeding.

Wang et al. performed caudal block with 20 mL of 1.2% lidocaine and reported a high complication rate [6]. Comparing with our results, the pain score of transrectal prostate biopsy was lower than ours. However, the pain score for the caudal block itself was also quite low, so caution should be taken when interpreting the results. Since they inserted gauze into the anus after prostate biopsy in all patients, the incidence of rectal bleeding was unknown. However, the incidence of other complications, such as urinary retention and urinary tract infection, was higher than that in our study [6]. Pasali et al. performed caudal block with 15 mL of 2% lidocaine. The prostate biopsy pain scores were similar to the results in our study. However, because the pain score of the caudal block itself was high, caution is required when interpreting pain control. Importantly, although details are unknown, the rate of rectal bleeding lasting more than 48 h after transrectal prostate biopsy was reported to be 7.5% [16]. A comprehensive review of previous reports suggests that a higher dose of 1% lidocaine in the caudal block for transrectal prostate biopsy may provide better pain control, but may increase the frequency of complications. Therefore, instead of blindly aiming for perfect pain control with a caudal block, the appropriate balance between pain control and complications should be investigated. In this study, it was expected that the 5 mL group would have a lower incidence of complications, but pain control would be insufficient. However, the results of this study showed that while pain control was comparable, the frequency and severity of rectal bleeding was significantly lower in the 5 mL group than in the 10 mL group. Therefore, it was suggested that 5 mL of 1% lidocaine for caudal block in prostate biopsy may be more appropriate than 10 mL.

If the amount of 1% lidocaine in a caudal block changes the pain and complication frequency during prostate biopsy, as suggested by previous reports [6, 7, 16, 17], then adjusting the amount of 1% lidocaine may enable better control of pain and complications. Therefore, it is important to consider the optimal dose of 1% lidocaine for caudal block during prostate biopsy. Our results theoretically suggest that pain control and complication frequency may have an inverse relationship [13, 14]. In other words, while a large amount of anesthesia may result in better pain control, it may also increase the frequency of complications. On the other hand, if the amount of anesthesia is low, complications may occur less frequently, but pain control may be insufficient. Therefore, a balance between pain control and complication control is important, but there are no reports comparing the amount of anesthesia for caudal block in prostate biopsy. Since we have customarily used two doses of 1% lidocaine for caudal block during prostate biopsy, it is appropriate to compare and examine pain control and complication frequency depending on the difference in anesthesia dose. We believe our results can provide an important reference when considering the amount of anesthesia.

In Europe, the primary anesthesia method currently used for transrectal prostate needle biopsy is transrectal ultrasound-guided prostate biopsy with PNB (TRUS-PNB) because it can successfully reduce the pain of prostate biopsy [18]. However, TRUS-PNB requires transrectal puncture for anesthetic administration, which may increase the risk of infection. Caudal block is an anesthesia method that does not require transrectal puncture and may potentially reduce the risk of infection compared to TRUS-PNB. Recently, a new minimally invasive and safe pain control method called the infiltration free local anesthesia (INFLATE) technique has been reported [19]. This method is a pain control method that uses transcutaneous electrical nerve stimulation and does not require transrectal puncture. Therefore, the INFLATE technique, such as the caudal block, can potentially reduce the risk of infection compared to TRUS-PNB [19]. The INFLATE technique has already been used to treat chronic prostatitis and pelvic pain syndrome, and its effectiveness has been reported [19, 20]. Therefore, the INFLATE technique may be a new pain management method for transrectal prostate needle biopsy that is expected to become popular in the future.

This study has several limitations. In this study, the lidocaine dose was determined by the day of the week the patient requested a prostate biopsy. Therefore, this allocation method lacks randomization and may introduce bias into the results of this study. In addition, this study is retrospective and has inherent limitations, such as reliance on existing medical records, incomplete data, and potential recall bias. Prospective RCTs are preferred to establish causality and reduce bias, and a more precise evaluation of the causal relationship between lidocaine dosage and outcome. Although we performed analysis using propensity-score matching, it was difficult to collect accurate information about patients' preexisting conditions and medical staff's experience, and the results of this study may be biased. In this study, 12 doctors performed caudal blocks and prostate biopsies. Therefore, there may be variations in the caudal block and prostate biopsy techniques. Although it is desirable to analyze results for each physician to reduce procedure bias, it has been difficult to calculate the exact number of cases each physician has experienced with caudal block and prostate biopsy. In addition, this study was conducted only at our hospital in Japan, which introduces various biases that may limit the generalizability of the study results. Characteristics of Japanese people include a smaller physique compared to Westerners, less obesity, and a lower BMI. Therefore, we believe that it is necessary to carefully consider whether the same results as this study can be obtained in other populations. However, the possibility that rectal bleeding may increase if the amount of anesthetic is increased is also considered to be true, and this may be helpful when considering the appropriate amount of anesthesia for caudal block. In addition, in this study, the dose-response relationship between 1% lidocaine dosage, pain management, and complication frequency was only compared between the 5 mL and 10 mL groups, and no further detailed examination was performed. Therefore, the results of this study are only a comparison between 5 and 10 mL, and it is not clear whether a similar relationship would be found with other 1% lidocaine doses. If we could collect data of other doses of 1% lidocaine, we would be able to gain a more comprehensive understanding. As mentioned above, this study contained a variety of potential biases and limitations, so a causal relationship cannot be completely established from the results of this study alone. Further research, particularly through more detailed comparative analysis or meta-analysis, is needed.

In conclusion, transrectal prostate biopsy using a caudal block with a low dose of 5 mL of 1% lidocaine provided comparable analgesic control to 10 mL of 1% lidocaine. Additionally, rectal bleeding, a complication of prostate biopsy, was less frequent and less severe with the lower dose of 5 mL compared to the traditional 10 mL dose. Despite various biases, these results suggest that 5 mL of 1% lidocaine may be a more appropriate dose for a caudal block during transrectal prostate biopsy than 10 mL.

## Figures and Tables

**Table 1:** Patient characteristics.

	*N* = 869 Median (range) or *N*
Age (years)	74 (46–95)
Height (cm)	165.7 (135.7–187.4)
Weight (kg)	64.1 (33.7–109.0)
BMI (kg/m^2^)	23.5 (13.3–37.6)
PSA (ng/mL)	8.32 (0.47–8600)
Prostate volume (mL)	38.0 (4.67–398)
The dosage of 1% lidocaine 5 mL 10 mL	466 403

BMI, body mass index; PSA, prostate-specific antigen.

**Table 2:** Comparison of patient characteristics with propensity-score matching.

	Prematching				Postmatching			
	1% lidocaine 5 mL (*n* = 466)	1% lidocaine 10 mL (*n* = 403)	*p* value	Standardized difference	1% lidocaine 5 mL (*n* = 395)	1% lidocaine 10 mL (*n* = 395)	*p* value	Standardized difference
Age (years), median (IQR)	74 (67–79)	74 (68–79)	0.67	0.024	74 (67–79)	73 (68–79)	0.89	0.012
Height (cm), median (IQR)	165.4 (161.0–169.6)	166.0 (162.0–170.0)	0.44	0.059	165.7 (161.5–169.8)	166.0 (161.9–169.7)	0.92	0.015
Weight (kg), median (IQR)	64.7 (57.5–70.63)	63.9 (57.7–70.0)	0.68	0.029	65.0 (57.7–70.5)	64 (57.8–70.0)	0.74	0.019
BMI (kg/m^2^), median (IQR)	23.7 (21.6–25.4)	23.3 (21.4–24.9)	0.33	0.062	23.7 (21.5–25.2)	23.3 (21.5–24.9)	0.68	0.027
PSA (ng/mL), median (IQR)	8.48 (5.8–13.2)	8.09 (5.7–15.6)	0.59	0.037	8.56 (5.89–13.0)	8.16 (5.70–15.72)	0.95	0.0042
Prostate volume (mL), median (IQR)	37.4 (27.7–55.0)	38.7 (28.0–53.0)	0.54	0.042	36.9 (27.2–54.6)	38.7 (28.0–53.0)	0.98	0.0012

Age, height, weight, BMI, PSA, and prostate volume were analyzed using the Mann–Whitney *U* test. BMI, body mass index; PSA, prostate-specific antigen; IQR, interquartile range.

**Table 3:** Cancer diagnosis rate of transrectal prostate biopsy.

	1% lidocaine 5 mL (*n* = 395)	1% lidocaine 10 mL (*n* = 395)	*p* value
c T-stage^†^ T1	26 (12.4%)	35 (16.4%)	0.24
T2	145 (69.1%)	132 (62.0%)	0.13
T3	38 (18.1%)	36 (16.9%)	0.75
T4	1 (0.48%)	10 (4.7%)	0.006^*∗∗*^
Cancer diagnosis rate (%)	53.1 (210/395)	53.9 (213/395)	0.83

^†^Data expressed as *n* (%). cT-stage and cancer diagnosis rate were analyzed using the chi-squared test. ^*∗∗*^*p* < 0.01.

**Table 4:** Results of pain scale score evaluation for caudal block, prostate biopsy, and anal sphincter tonus relaxation.

	1% lidocaine, 5 mL (*n* = 395)	1% lidocaine, 10 mL (*n* = 395)	*p* value
NRS pain score of caudal block, median (IQR)	2.0 (2.0–5.0)	2.0 (2.0–5.0)	0.91
NRS pain score of prostate biopsy, median (IQR)	2.0 (2.0–5.0)	2.0 (2.0–4.0)	0.12
Anal sphincter tonus score, median (IQR)	1.0 (0–1.0)	1.0 (0–1.0)	0.28

NRS pain score and anal sphincter tonus score were analyzed using the Mann–Whitney *U* test. NRS, numerical rating scale; IQR, interquartile range.

**Table 5:** Early complications of transrectal prostate biopsy.

	1% lidocaine, 5 mL (*n* = 395)	1% lidocaine, 10 mL (*n* = 395)	*p* value
Rectal bleeding (%)	59.7 (236/395)	67.8 (268/395)	0.018^*∗*^
Rectal bleeding score	0.77 ± 0.77	0.93 ± 0.83	0.0036^*∗∗*^
Urinary retention (%)	3.5 (14/395)	4.8 (19/395)	0.38
Urinary tract infection (%)	1.3 (5/395)	1.5 (6/395)	0.76
Vagal reflex (%)	1.5 (6/395)	2.8 (11/395)	0.22
Hematuria (%)	4.6 (18/395)	2.8 (11/395)	0.19

Rectal bleeding score was analyzed using the Mann–Whitney *U* test. ^*∗∗*^*p* < 0.01 using the Mann–Whitney *U* test. The incidences of complications were analyzed using the chi-square test. ^*∗*^*p* < 0.05 using the chi-squared test.

**Table 6:** Univariate and multivariate logistic regression analysis of patient characteristics exploring factors related to rectal bleeding.

	Univariate		Multivariate	
	Odds ratio (95% CI)	*p* value	Odds ratio (95% CI)	*p* value
Age (years)	1.00 (0.98–1.02)	0.81	1.00 (0.98–1.02)	0.71
Height (cm)	1.00 (0.98–1.03)	0.75	1.06 (0.90–1.26)	0.48
Weight (kg)	1.00 (0.99–1.02)	0.87	0.93 (0.75–1.15)	0.52
BMI (kg/m^2^)	1.00 (0.95–1.05)	0.94	1.21 (0.67–2.19)	0.52
PSA (ng/mL)	1.00 (0.99–1.00)	0.99	1.00 (0.99–1.00)	0.98
Prostate volume (mL)	1.00 (0.99–1.01)	0.33	1.00 (0.99–1.01)	0.34
Dosage of 1% lidocaine (5 ml vs. 10 ml)	1.42 (1.06–1.90)	0.018^*∗*^	1.42 (1.06–1.90)	0.019^*∗*^

^*∗*^ *p* < 0.05 using univariate and multivariate logistic regression analysis. BMI, body mass index; PSA, prostate-specific antigen; IQR, interquartile range.

**Table 7:** Relationship between 1% lidocaine dosage for caudal block and rectal bleeding score.

	1% lidocaine, 5 mL	1% lidocaine, 10 mL	Total
Rectal bleeding score 0	159	127	286
Rectal bleeding score 1	184	191	375
Rectal bleeding score 2	37	53	90
Rectal bleeding score 3	15	24	39
Total	395	395	790

## Data Availability

The data that support the findings of this study are available from the corresponding author upon reasonable request.
